# Assessment of the potential for gene flow from transgenic maize (*Zea mays* L.) to eastern gamagrass (*Tripsacum dactyloides* L.)

**DOI:** 10.1007/s11248-017-0020-7

**Published:** 2017-05-02

**Authors:** Moon-Sub Lee, Eric K. Anderson, Duška Stojšin, Marc A. McPherson, Baltazar Baltazar, Michael J. Horak, Juan Manuel de la Fuente, Kunsheng Wu, James H. Crowley, A. Lane Rayburn, D. K. Lee

**Affiliations:** 1 Department of Crop Sciences, University of Illinois at Urbana-Champaign, 1102 S. Goodwin Ave., Urbana, IL 61801 USA; 2 Monsanto Company, 800 North Lindbergh Blvd., St. Louis, MO 63167 USA; 3 Monsanto Company, Park Plaza Torre II, 504 Javier Barros Sierra Ave., Col. Santa Fe, Del. Alvaro Obregon, CP 01210 Mexico, DF Mexico; 4 Monsanto Company, 700 Chesterfield Parkway W., St. Louis, MO 63017 USA

**Keywords:** Eastern gamagrass, Transgenic maize, Gene flow, Glyphosate-tolerance

## Abstract

Eastern gamagrass (*Tripsacum dactyloides* L.) belongs to the same tribe of the Poaceae family as maize (*Zea mays* L.) and grows naturally in the same region where maize is commercially produced in the USA. Although no evidence exists of gene flow from maize to eastern gamagrass in nature, experimental crosses between the two species were produced using specific techniques. As part of environmental risk assessment, the possibility of transgene flow from maize to eastern gamagrass populations in nature was evaluated with the objectives: (1) to assess the seeds of eastern gamagrass populations naturally growing near commercial maize fields for the presence of a transgenic glyphosate-tolerance gene (*cp4 epsps*) that would indicate cross-pollination between the two species, and (2) to evaluate the possibility of interspecific hybridization between transgenic maize used as male parent and eastern gamagrass used as female parent. A total of 46,643 seeds from 54 eastern gamagrass populations collected in proximity of maize fields in Illinois, USA were planted in a field in 2014 and 2015. Emerged seedlings were treated with glyphosate herbicide and assessed for survival. An additional 48,000 seeds from the same 54 eastern gamagrass populations were tested for the presence of the *cp4 epsps* transgene markers using TaqMan^®^ PCR method. The results from these trials showed that no seedlings survived the herbicide treatment and no seed indicated presence of the herbicide tolerant *cp4 epsps* transgene, even though these eastern gamagrass populations were exposed to glyphosate-tolerant maize pollen for years. Furthermore, no interspecific hybrid seeds were produced from 135 hand-pollination attempts involving 1529 eastern gamagrass spikelets exposed to maize pollen. Together, these results indicate that there is no evidence of gene flow from maize to eastern gamagrass in natural habitats. The outcome of this study should be taken in consideration when assessing for environmental risks regarding the consequence of gene flow from transgenic maize to its wild relatives.

**Electronic supplementary material:**

The online version of this article (doi:10.1007/s11248-017-0020-7) contains supplementary material, which is available to authorized users.

## Introduction

A total of 322 transgenic crop products have been approved for cultivation worldwide since its first commercialization in 1996 (ISAAA 2016). In the USA alone, 70.9 million hectares were planted with transgenic crops in 2015 with high adoption rates for maize (92%), soybean (94%) and cotton (94%) (James, 2015). Gene flow as a result of outcrossing is one of the considerations of environmental risk assessment prior to commercialization of transgenic crops. The likelihood and consequences of potential pollen-mediated gene flow from transgenic crops to their wild relatives is assessed to assure that a transgene will not contribute a competitive advantage to the receiving species that might result in increased weediness and invasiveness, or in a decrease in biodiversity and disruption of ecological equilibrium (Tiedje et al. 1989; Williamson 1994; Ellstrand 2001; Gewin 2003).

When assessing for risk associated with potential pollen-mediated gene flow in nature from transgenic maize (*Zea mays* L.) to eastern gamagrass (*Tripsacum dactyloides* L.), several factors need to be considered such as shared habitats, exposure to outcrossing and sexual compatibility between the two species.

### Shared habitats between eastern gamagrass and maize

Eastern gamagrass is an extremely diverse warm-season, perennial grass that grows naturally in prairie remnants and is native to North and South America. The majority of *Tripsacum* species have been observed in Mexico (Newell and de Wet 1974; de Wet et al. 1982; Li et al. 1999) with their habitat extending from 42°N in the USA to 24°S in South America (de Wet et al. 1982). Besides natural populations, diverse cultivars of eastern gamagrass are commonly used for forage production, erosion control and wildlife plantings in the USA (Kindiger and Dewald 1997; Springer and Dewald 2004). The regions in North and South America where eastern gamagrass grows are also significant maize production areas with a high proportion of commercially grown transgenic maize.

### Exposure to outcrossing

In 2015, the area planted with transgenic maize was 33.1 million hectares in the USA, 13.1 million hectares in Brazil and 2.9 million hectares in Argentina, which jointly represent a major portion of 53.6 million hectares of transgenic maize grown globally (James 2015).

In the USA, transgenic maize with herbicide-tolerance has been grown adjacent to eastern gamagrass natural populations for years, as herbicide-tolerant maize made up 7% of the USA production in 2000 (Fernandez-Cornejo et al. 2014) and increased to 96% by 2015 (James 2015). Similarly, most transgenic maize grown in Brazil and Argentina in 2015 contained herbicide tolerance traits: 63 and 76%, respectively (James 2015).

Commercial maize hybrids with tolerance to glyphosate (an active ingredient in the *Roundup* ^®^ 1 agricultural herbicides) include NK603 and MON88017 transgenes. The maize hybrids containing the NK603 transgene have been commercially grown in the USA since 2001, whereas those with MON88017 transgene have been cultivated since 2005. Both transgenes were developed by Monsanto Co. (St. Louis, MO, USA) and for both, the herbicide-tolerance is due to a production of the 5-enolpyruvylshikimate-3-phosphate synthase protein from *Agrobacterium* sp. strain CP4 (CP4 EPSPS) that confers tolerance to the herbicide glyphosate. The long duration of these transgenic maize products in the market and their high adoption rate by farmers have resulted in a very high exposure of eastern gamagrass populations to maize pollen with the *cp4 epsps* transgene in the USA Corn Belt region.

### Sexual compatibility

Eastern gamagrass is a wild relative of maize and they both belong to the tribe Andropogoneae of the Poaceae family. Genetic diversity of eastern gamagrass is exemplified by different ploidy levels observed among natural populations. The three major variants are: diploid (2n = 2x = 36), triploid (2n = 3x = 54) and tetraploid (2n = 4x = 72) cytotypes (Newell and de Wet 1974; de Wet et al. 1982). Pentaploid (2n = 5x = 90) and hexaploid (2n = 6x = 108) cytotypes have also been reported (Newell and de Wet 1974; Farquharson 1955; Kindiger and Dewald 1997). The reproductive biology of gamagrass appears to be correlated with ploidy level as diploid populations reproduce sexually, whereas polyploid populations predominantly reproduce through facultative apomixes and pseudogamy (Farquharson 1955; Newell and de Wet 1974; Burson et al. 1990; Sherman et al. 1991; Dewald and Kindiger 1994). The majority of gamagrass populations that grow naturally through the central and eastern USA are diploid and tetraploid cytotypes (Newell and de Wet 1974). Compared to eastern gamagrass (where ploidy levels range from 2x to 6x with corresponding chromosome number range from 36 to 108), maize is a diploid species with a stable chromosome number (2n = 2x = 20) and sexual reproduction.

Outcrossing between species of different genera can occur (Raybould and Gray 1993), but it is generally lower in frequency compared to outcrossing within the same species or between species of the same genus due to a lower level of genetic compatibility. Because *Tripsacum* and *Zea* are closely related genomes, eastern gamagrass has been considered as a possible source of favorable and diverse genes for maize improvement (Newell and de Wet 1974; de Wet et al. 1982; Li et al. 1999; de Wet and Harlan 1974; Leblanc et al. 1995; Reeves and Bockholt 1964; Duvick et al. 2006; Hajjar and Hodgkin 2007). *Tripsacum*’s wide tolerance to soil conditions, resistance to some common maize diseases and insect pests, favorable fatty acid composition and ability to reproduce through apomixis are examples of characteristics that make this species potentially valuable for maize breeding programs (Doebley 1983; Bernard and Jewell 1985; Leblanc et al. 1995; Gurney et al. 2003; Duvick et al. 2006; Belova et al. 2010).

Collins and Kempton (1914, 1916) attempted the first interspecific hybridization between maize and eastern gamagrass, but were not successful. Mangelsdorf and Reeves (1931) produced the first true, interspecific hybrids with maize used as the maternal parent and eastern gamagrass (both diploid and tetraploid cytotypes) as the paternal parent. However, the success of artificial hybridization was accomplished only with special techniques (Beadle 1980) such as removing husk leaves from maize ears, and cutting the maize silks to accommodate for shorter eastern gamagrass pollen tubes (Mangelsdorf and Reeves 1931; Bernard and Jewell 1985). The shorter maize silks eliminated one of the cross-incompatibility obstacles (lack of growth support for the eastern gamagrass pollen tube within the maize silk), however there are other late-acting barriers which prevent formation of hybrid zygote in most intergeneric crosses between grasses (Dresselhaus et al. 2011). Utilization of embryo culture techniques (Randolph 1970; James 1979; Bernard and Jewell 1985) was also needed to generate these experimental hybrids between eastern gamagrass and maize. The resulting hybrid seeds were obtained at very low frequency, showed variable and highly abnormal development resulting in reduced germination and low seedling survival (Mangelsdorf and Reeves 1931). The seedlings generally exhibited retarded development with no roots or significantly delayed root system growth (Bernard and Jewell 1985). Hybrid plants that survived to flowering had complete male sterility (lack of anthers extrusion, aborted pollen grains), reduced female sterility, variability in chromosome number within the cells of the same tissue, and erratic and progressive chromosome elimination in subsequent generations (Mangelsdorf and Reeves 1931; James, 1979; Beadle 1980; Leblanc et al. 1995; Graminelli et al. 1996; Randolph 1970).

To overcome hybrid sterility, the interspecific hybrid plants needed to be backcrossed to maize producing offspring that exhibited a rapid loss of *Tripsacum* chromosomes and partial to high sterility even after multiple backcross generations (de Wet and Harlan 1972; Graminelli et al. 1996; Belova et al. 2010). In some studies, none of the backcrosses were successful in producing fertile plants (James 1979). This genomic instability and sterility of hybrids between maize and eastern gamagrass have been some of the factors that limited the usefulness of eastern gamagrass in maize breeding programs.

The hybrids reported in literature were generally accomplished with maize used as the female parent (Mangelsdorf and Reeves 1931; James 1979; Bernard and Jewell 1985; Leblanc et al. 1995; Duvick et al. 2006; Belova et al. 2010). When assessing the environmental risk of gene flow from transgenic maize to eastern gamagrass, the crosses where eastern gamagrass is used as a female parent would be most informative. Mangelsdorf and Reeves (1931) tried to produce interspecific hybrids using eastern gamagrass as a female parent, but were not successful. A couple of researchers reported producing experimental hybrids with eastern gamagrass used as pollen recipient (Farquharson 1957; de Wet et al. 1984). However most of the resulting seeds failed to germinate (Farquharson 1957). Those that germinated had abnormal seedlings that did not survive. Some seedlings were not true hybrids as they had no maize chromosome which indicated lack of fertilization (de Wet et al. 1984; Farquharson 1957). A few plants that were identified as true hybrids in these studies were not mentioned in subsequent research publications. In a comprehensive review of genetic research on *Tripsacum*, Blakey et al. (2007) summarized extensive hybridization effort where maize was used as a female parent, without a mention of any research with hybrids resulting from maize as a male parent.

Most experts agree that *Tripsacum* and maize do not naturally cross-pollinate in spite of growing in close proximity for centuries (Mangelsdorf and Reeves 1931; Randolph 1970; Beadle 1980; Dresselhaus et al. 2011). No evidence was found for ongoing natural gene introgression between maize and *Tripsacum* although careful and extensive research was conducted across large number of populations from Mexico, Guatemala and South America (de Wet and Harlan 1978; de Wet et al. 1981). The severely limited fertility demonstrated in experimental hybrids presents a significant biological barrier to gene flow between maize and eastern gamagrass (Eubanks 1994). However, the possibility of obtaining experimental interspecific hybrids between maize and eastern gamagrass using special greenhouse and laboratory techniques has raised the question about the probability of natural outcrossing between these two species to the attention of risk assessors/evaluators. This ambiguity prompted the Mexican Ministries of Agriculture and Environment to include areas where *Tripsacum* grows naturally as consideration for the isolation areas defined in the current version of the Agreement for Maize Center of Origin to prevent potential flow of transgenes from maize to *Tripsacum* (SAGARPA 2012).

The present research was conducted to contribute to a better scientific understanding of a possibility of gene flow from maize to eastern gamagrass in nature, and to assist decision-makers in the assessment of environmental risks regarding the likelihood and potential consequence of gene flow from transgenic maize to eastern gamagrass. The objectives of this study were: (1) to assess if the gene flow occurs in nature from cultivated transgenic maize to eastern gamagrass and (2) to evaluate the possibility of interspecific hybridization between transgenic maize used as male parent and eastern gamagrass used as female parent when aided by hand-pollination under greenhouse conditions. To our knowledge this is the first study to evaluate the potential of pollen-mediated gene flow from transgenic maize to natural populations of eastern gamagrass. The results obtained in this study are relevant to environmental risk assessment of transgenic maize in regions where maize and eastern gamagrass coexist.

## Materials and methods

### Assessment of gene flow from glyphosate-tolerant maize to eastern gamagrass

#### Plant material

Seeds collected from natural populations of eastern gamagrass growing in Illinois, USA were used in this study. At each location, seeds were collected within 50 m of a field where at least 40 ha of transgenic maize was cultivated in the same year. Furthermore, the area where collections were made was within the region of major commercial maize production ensuring long period of eastern gamagrass exposure to transgenic maize pollen. A total of 54 eastern gamagrass populations were sampled from August through September in both 2013 and 2014.

The populations were collected from 43 different Illinois locations. At some locations, more than one population was collected if they were found in close proximity and if they had some observed phenotypic differences that would justify classifying them as different populations (e.g., EG 211 and EG 212; EG 124 and EG 125). Also, populations were given unique names if they were collected at the same location, but in different years (e.g., EG 117 and EG 214). In most cases, only a single population of eastern gamagrass was found at a given location and in a given year.

A total of 73,930 seeds from 26 populations and 66,407 seeds from 28 populations were collected in 2013 and 2014, respectively (Table 1). Collected seed was stored at 4 °C until field screening and molecular assay.

**Table 1:** Details regarding seed quantity and locations in Illinois, USA where seeds of eastern gamagrass populations (*T. dactyloides* L.) were collected

Collection year	Accession ID^a^	Longitude	Latitude	Total number of collected seed	Seed number for molecular screen	Seed number for field screen
2013	EG 101	38°34′N	88°59′W	1143	600	540
	EG 102	38°20′N	89°17′W	1953	1000	950
	EG 103	38°21′N	89°38′W	1373	700	670
	EG 104	38° 22′ N	89°46′W	1993	1000	990
	EG 105	38°39′N	89°48′W	1543	800	740
	EG 106	38°43′N	89°44′W	1953	1000	950
	EG 107	38°38′N	89°06′W	1183	600	580
	EG 108	38°38′N	89°06′W	1287	600	580
	EG 109	38°40′N	89°06′W	2681	1000	1000
	EG 110	38°45′N	89°05′W	1488	700	740
	EG 111	38°44′N	89°05′W	1208	600	600
	EG 112	38°41′N	89°06′W	1002	500	500
	EG 113	38°38′N	89°06′W	1826	900	910
	EG 114	38°32′N	89°11′W	1405	700	700
	EG 115	38°32′N	89°21′W	1683	800	840
	EG 116	38°32′N	89°24′W	1963	1000	960
	EG 117	38°32′N	89°26′W	2103	1000	1000
	EG 118	38°36′N	89°26′W	1823	900	910
	EG 119	40°05′N	88°13′W	1217	600	600
	EG 120	40°04′N	88°13′W	260	100	130
	EG 121	40°04′N	88°13′W	2286	1000	1000
	EG 122	40°04′N	88°13′W	1163	600	560
	EG 123	40°04′N	88°13′W	1410	700	700
	EG 124	40°08′N	88°22′W	35,000	2000	3000
	EG 125	40°08′N	88°22′W	1882	1000	800
	EG 126	40°08′N	88°23′W	1102	500	500
Subtotal				73,930	20,900	21,450
2014	EG 201	38°36′N	89°08′W	2016	1000	1000
	EG 202	38°37′N	89°09′W	2008	1000	1000
	EG 203	38°38′N	89°13′W	2145	1000	1000
	EG 204	38°39′N	89°15′W	2031	1000	1000
	EG 205	38°40′N	89°13′W	2011	1000	1000
	EG 206	38°45′N	89°11′W	2074	1000	1000
	EG 207	38°47′N	89°09′W	2007	1000	1000
	EG 208	38°56′N	89°05′W	2214	1000	1000
	EG 209	38°38′N	89°06′W	2361	1000	1000
	EG 210	38°30′N	89°06′W	2140	1000	1000
	EG 211	38°32′N	89°21′W	2081	1000	1000
	EG 212	38°32′N	89°21′W	2150	1000	1000
	EG 213	38°32′N	89°23′W	2207	1000	1000
	EG 214	38°32′N	89°26′W	2182	1000	1000
	EG 215	38°35′N	89°23′W	2014	1000	1000
	EG 216	38°38′N	89°22′W	2413	1000	1000
	EG 217	38°66′N	90°17′W	616	300	133
	EG 218	38°76′N	89°81′W	1663	800	429
	EG 219	38°73′N	89°37′W	1968	1000	445
	EG 220	38°55′N	89°33′W	2108	1000	573
	EG 221	38°75′N	89°91′W	2751	1000	1000
	EG 222	38°72′N	89°91′W	2766	1000	1000
	EG 223	38°67′N	90°07′W	2969	1000	1000
	EG 224	38°62′N	89°38′W	3009	1000	1000
	EG 225	38°76′N	89°86′W	3097	1000	1000
	EG 226	38°59′N	89°37′W	3108	1000	1000
	EG 227	38°75′N	89°91′W	3936	1000	1000
	EG 228	38°53′N	89°35′W	4362	1000	613
Subtotal				66,407	27,100	25,193
Total				140,337	48,000	46,643

^a^EG 101- EG 228 were designations for 54 eastern gamagrass populations

#### Field screen

A field experiment was conducted in 2014 and 2015 to determine whether natural eastern gamagrass populations exhibited tolerance to the glyphosate herbicide (S-Table 1). A total of 21,450 seeds representing 26 natural populations from 2013 collection and 25,193 seeds collected from 28 natural populations in 2014 were used in the experiment. Since eastern gamagrass is slow and difficult to establish from seed, stratification was conducted prior to planting to improve germination and emergence rate (USDA NRCS 2015). Seed stratification was conducted by following these steps: (1) collected seeds were immersed in water with fungicide (ApronMaxx RTA, Syngenta Crop Production, Inc., Greenboro, NC, USA) for 24 h and (2) seed was drained, placed in sealed plastic bags and maintained at 10 °C for 60 days. Stratified seeds were planted in a Flanagan silt loam soil at the University of Illinois Research Farm, Urbana, IL (40°04′N, 88°13′W). Planting was conducted on April 23, 2014 and May 18, 2015 at 3 cm depth using a two-row plot planter. Inter-row spacing was 76 cm. Conventional and glyphosate-tolerant maize hybrids were also planted as control checks. Seeds from each eastern gamagrass population and maize hybrid were planted in single rows, 25 m in length. Seedling emergence was recorded weekly for 4 weeks after planting. When seedlings of eastern gamagrass reached the 3–4 leaf stage, glyphosate was applied at 334 g acid equivalent (a.e.) ha^−1^ rate (*Roundup* ^®^ *Powermax*, Monsanto Company, St. Louis, MO, USA) with 2% v/v ammonium sulfate as an additive. Maize plants were at the 6–8 leaf stage at the time of herbicide application. Plant mortality was evaluated weekly over a 2-week period. Complete mortality of conventional maize indicated that the glyphosate application was conducted correctly.

Seedlings of eastern gamagrass that emerged after the glyphosate application were transplanted into 2-L pots and allowed to grow outdoors until they reached the 3–4 leaf stage. Glyphosate was applied at the previously described rate using a CO_2_ backpack sprayer calibrated to deliver 168 L ha^−1^ at 220 kPa through a two-nozzle spray boom. Plant mortality was observed weekly over a 2-week period.

#### Development and validation of cp4 epsps marker

Seeds of eastern gamagrass, maize with the CP4 EPSPS trait, that confers tolerance to glyphosate, (the NK603 and MON88017 products) and conventional maize (without the transgenic CP4 EPSPS trait) were each ground on dry ice in separate batches. Material from these ground samples were used to develop a dilution series and simulate an eastern gamagrass sample with CP4 EPSPS trait from NK603 and MON88017. This method development step was conducted to ensure that even a small quantity of transgene presence could be detected in eastern gamagrass. As samples of 25, 50, 100, 200 and 400 bulked seeds were considered, the ground maize material was mixed with ground eastern gamagrass material in 1:25, 1:50, 1:100, 1:200 and 1:400 ratio. The weight used to represent gamagrass seed was based on minimum weight evaluated for 12 eastern gamagrass populations (data not shown). In addition, ground seed samples of NK603 maize, MON88017 maize and eastern gamagrass were each produced as controls. For marker development and validation, DNA was extracted from ground seed using Qiagen Plant DNeasy kit (Qiagen Inc., Hilden, Germany).

Endpoint TaqMan^®^ PCR was used for detection of the presence of *cp4 epsps* transgene in eastern gamagrass samples. Endpoint TaqMan^®^ markers were first designed and validated in the Marker Discovery and Development laboratory in Monsanto Co. (St. Louis, MO, USA) and then the methodology was transferred to Eurofins BioDiagnostics Inc. (River Falls, WI, USA) for use in a large-scale screening. To ensure that the marker used in this study was sensitive enough to detect at least one seed containing *cp4 epsps* transgene in a pool of seeds, nine different primers/probes combinations specific for *cp4 epsps* were designed and tested with three different maize internal controls as duplex markers. The DNA probe for *cp4 epsps* was labeled a FAM™ dye, while the DNA internal controls were labeled the VIC^®^ dye (Applied Biosystems, Waltham, MA, USA). The marker selected for use in large-scale screening consisted of the following components:

- Forward primer, SQ23390, 5′-ACGATTTCGACAGCACCTTCA-3′
- Reverse primer, SQ23391, 5′-GTCACCGTCTTCCGATTTCAC-3′
- Probe, PB50320, 5′-6FAM-TCGGCGACGCCTC-3′-MGB
- Internal control forward primer, SQ1241, 5′-GCCTGCCGCAGACCAA-3′
- Internal control reverse primer, SQ1242, 5′-CAATGCAGAGCTCAGCTTCATC-3′
- Internal control probe, PB50323, 5′-VIC-TCCAGTACGTGCAGTCCCTCCTCCCT-3′-TAMRA.

**Fig. 1:**
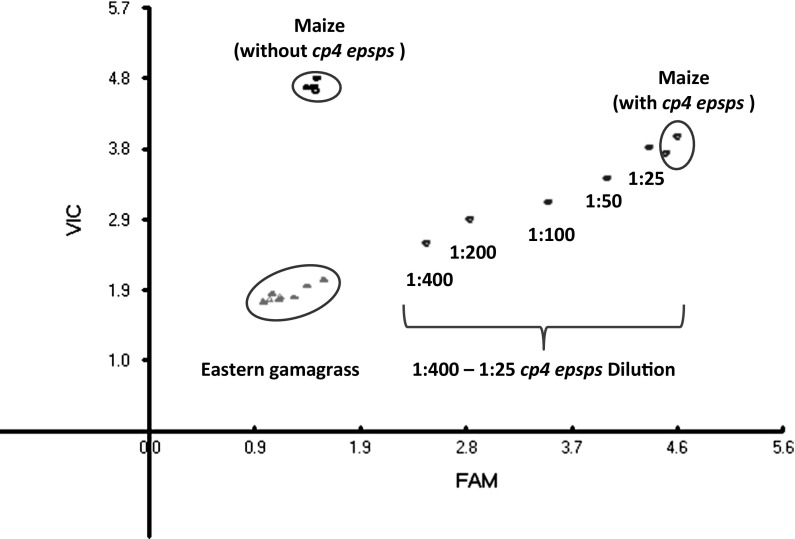
Genotyping plot for CP4 EPSPS trait using Endpoint TaqMan^®^ PCR method (*cp4 epsps* marker with maize internal control). VIC and FAM represent the signal intensity ratios of each dye to a ROX dye standard added to each reaction. VIC dye-labeled DNA probe identifies the maize internal standard gene and FAM identifies the *cp4 epsps* transgene (NK603 product). The figure does not contain a control without the DNA template, as it was not needed in this method development experiment

A maize internal control was used that targeted the bnlg1079 locus (Andorf et al. 2010), The control was maize specific and was not cross-amplified in eastern gamagrass. Samples containing *cp4 epsps* were designated as positive, and those not containing the transgene were designated as negative. This TaqMan^®^ assay was able to distinguish the three clusters for (1) eastern gamagrass, (2) maize control without the *cp4 epsps* transgene, and (3) maize samples with the *cp4 epsps* transgene (Fig. 1). The TaqMan^®^ assay was able to detect the *cp4 epsps* transgene in both NK603 and MON88017.

An eastern gamagrass marker was developed for use as a control to assure that the eastern gamagrass DNA samples used in this study were assayable. The eastern gamagrass marker detects a single copy EST sequence, GenBank Accession AY789601 (FL2 gene), and also detects maize DNA due to sequence homology between these two species. The FL2 gene marker allowed for the same marker to be used as an internal standard to confirm DNA extraction quality for both eastern gamagrass samples and maize control samples. The eastern gamagrass marker was comprised of:

- Forward primer, SQ50935, 5′-GAGGAGGAAGAAGGAGCTGAGG-3′
- Reverse primer, SQ50936, 5′-CCTCGTCTCCGTCGTTCGT-3′
- Probe, PB50321, 5′-TGGACGTGCTGGACGA-3′.

Genotyping for marker development was done in a 5 µl reaction consisting of 2.5 µl GTXpress master mix (Applied Biosystems, Waltham, MA, USA), 0.02 µl each of the four primers at 100 µM concentration, 0.01 µl each of the two probes at 100 µM, 4 ng template DNA plus water to make up 5 µl. The PCR assay was carried on a GeneAmp PCR System 9700 Thermal Cycler (Applied Biosystems, Waltham, MA, USA) with the following temperature profile: 95 °C for 20 s; 35 cycles of 95 °C for 3 s and 60 °C for 20 s, and hold at 10 °C. The GTXpress master mix contains a ROX internal standard dye used to normalize each assay. The plate was read on a Tecan Safire Microplate reader (Tecan Group, Ltd., Männedorf, Switzerland).

A validation test was performed at Eurofins BioDiagnostics, Inc. (River Falls, WI, USA) to confirm that the laboratory was proficient to conduct the assay at their facility. A 96-well validation plate was prepared by Monsanto that contained 40 ground samples of 1:50 ratio of maize with MON88017 in eastern gamagrass and 40 samples of an eastern gamagrass control. The samples were randomized in a single blind study (blind to Eurofins BioDiagnostics). The results showed 0% false positive and 0% false negative readings, which was sufficient to deem the Eurofins BioDiagnostics laboratory proficient to run the assay on 50 seed pools of eastern gamagrass.

#### Molecular screen

For screening the collected eastern gamagrass populations for presence of CP4 EPSPS trait, on average 18 samples of 50 seeds each were prepared for each population and subjected to molecular assays. Each 50-seed sample was ground with dry ice, and stored at −80 °C until analysis. A total of 960 samples (48,000 seeds) from 54 populations were tested. Genomic DNA was extracted from 6 to 9 mg of each 50-seed pooled sample using the BDI Prime™ DNA extraction method. This method was developed at Eurofins BioDiagniostics as a commercially available service, using a silica filter based DNA extraction. The BDI Prime™ DNA extraction was developed based on the protocol described by Whitlock et al. (2008) with some modifications. Each 50-seed sample was subjected to amplification in 10 µl PCR reactions consisting of 5 µl Perfecta Custom Genotyping Toughmix master mix (Quanta Biosciences, Inc., Beverly, MA, USA), 0.1 µl each of the two CP4 EPSPS product primers at 100 µM concentration, 0.02 µl of the event probe at 100 µM, 12 ng template DNA plus proper amount of water to make up 10 µl. The endogenous reference assay was run as a separate assay using the same quantities as the *cp4 epsps* assay. Endpoint TaqMan^®^ PCR was performed on an ABI Veriti PCR Thermal Cycler (Thermo Fisher Scientific, Foster City, CA, USA) with the following temperature profile: 95 °C for 30 s; 35 cycles of 95 °C for 3 s and 60 °C for 20 s. The plate was read on a Molecular Devices Gemini XPS Microplate reader (Molecular Devices, Sunnyvale, CA, USA).

### Assessment of interspecific hybridization between maize and eastern gamagrass

#### Plant material

To make interspecific hybrids by hand-pollination in the greenhouse, rhizomes were collected from an eastern gamagrass field nursery at the University of Illinois at Urbana-Champaign in 2014 and 2015 from population EG 124. After a killing frost, eastern gamagrass rhizomes were unearthed and stored at 4 °C in a cold room. Eastern gamagrass rhizomes were transplanted into pots in the greenhouse. Since diploid eastern gamagrass has higher chances of sexual reproduction compared to a tetraploid cytotype, the ploidy level of the rhizomes was determined by flow cytometric analysis and confirmed by chromosome count.

#### Ploidy confirmation

Nuclear DNA content determination was conducted using a procedure slightly modified from Rayburn et al. (2005). Briefly, 2.5 cm of fresh young stems from eastern gamagrass and maize (used as an internal control), were co-homogenized and placed in a 20-ml beaker containing 10 ml extraction buffer and 200 µl 25% Triton X. The extraction buffer was composed of 13% v/v hexylene glycol, 10 mM Tris–HCl, and 10 mM MgCl_2_. The nuclear DNA content of the maize was calibrated at 5.14 pg using sorghum hybrid 84G62 with 1.74 pg/2C nuclei (Rayburn et al. 2005). The tissue was homogenized using a tissue grinder for 20 s at 4500 rpm, and the samples were filtered through a 50 nm filter into a test tube and maintained on ice. Following filtration, samples were centrifuged for 20 min at 2000 rpm at 4 °C. The supernatant was removed, and nuclei were re-suspended in 300 µl of PI stain (3% w/w polyethylene glycol 6000, 50 µg ml^−1^ PI, 180 U ml^−1^ RNase, 0.1% Triton X-100 in 4 mM citrate buffer). The solution was transferred to a 1.5-ml conical tube and incubated for 20 min at 37 °C. Following incubation, 300 µl of PI salt (3% PEG, 50 µg ml^−1^ PI, 0.1% Triton X-100 in 400 mM NaCl) was added to each sample. Samples were then briefly vortexed, placed on ice, and stored at 4 °C for at least 1 h. The analysis of relative DNA content was conducted with BD LSR flow cytometry (BD Biosciences, San Jose, CA, USA) equipped with a excitation wavelength of 488 nm and a 680/45 filter in the Flow Cytometry Laboratory (Biotechnology Center, University of Illinois at Urbana-Champaign, USA). Approximately 30,000 nuclei per sample were analyzed. The relative DNA content was estimated using the relative fluorescence of the sample divided by the relative fluorescence of the standard.

To confirm ploidy levels by chromosome count, actively growing root tips (about 2 cm in length) were collected from eastern gamagrass plants used for nuclear DNA content determination. Chromosome preparation was conducted according to Kim et al. (2010). The root tips, which were collected from plants transplanted into pots and maintained in the greenhouse, were pretreated with 20 ml of 0.05% 8-hydroxyquinoline for three hours and fixed at room temperature for at least one day in a 3:1 ratio of ethanol to acetic acid. The fixed root tips were rinsed in double distilled H_2_O, hydrolyzed in 5 N HCl for 45 min, and stained with Feulgen’s reagent for two hours. The root tips were then rinsed again with double distilled H_2_O and soaked in an enzyme solution (0.2 g Cellulysin and 0.1 g Macerase in 10 ml of 10 mM EDTA) for two hours. One drop of acetic acid was added to the root tips before squashing. A cover slide was placed over the tissue and then squashed with thumb pressure. The cells were imaged with a microscope at 60x magnification (Olympus BX61, Olympus Scientific Solutions Americas Corporation, Waltham, MA, USA) and photographed with a digital camera (Olympus U-CMAD3).

#### Interspecific and intraspecific pollinations

For the greenhouse experiment, a total of 20 and 32 diploid eastern gamagrass plants were used as female parents in 2014 and 2015, respectively. Maize plants homozygous for glyphosate-tolerant CP4 EPSPS trait (NK603) were used as male parents. To synchronize the flowering time of the two species, maize was planted at weekly intervals between June and November in 2014 and between March and July in 2015. When eastern gamagrass spikes emerged (and prior to pollen shed) the male portion of the flower was removed and the female portion was covered with a pollination bag. At anthesis, maize pollen was transferred to the stigmas of eastern gamagrass. The pollinated gamagrass spikes were then covered again with an isolation bag to protect against uncontrolled pollination (S-Fig. 1).

Hand pollination was performed within each species (i.e., eastern gamagrass and maize) as a control to test the success of the pollination procedure in each year. For eastern gamagrass, pollen from one eastern gamagrass plant was used to pollinate stigmas of another plant (sib-pollination). For maize, ears were bagged prior to silking, and tassels were bagged at anthesis the day before self-pollination was conducted.

Eastern gamagrass spikelets were harvested at maturity and each individual spikelet was examined for the presence of the seed. Every identified seed was tested for germination. Tissues from resulting seedlings and from the seed that failed to germinate were tested for the presence of *cp4 epsps*. The testing was conducted using QuickStix™ Strips for *cp4 epsps* Leaf and Seed (Envirologix Inc., Portland, MN, USA) for detection of the transgenic protein, as well as Endpoint TaqMan^®^ PCR assay described earlier for detection of the transgene.

## Results and discussion

### Assessment of gene flow from glyphosate-tolerant maize to eastern gamagrass

A total of 140,337 seeds representing 54 eastern gamagrass populations were collected from 43 diverse Illinois locations where it grows naturally in close proximity of maize fields (Table 1). The zygosity was not determined for these populations, with the exception of the EG 124 population which was comprised of both diploid and tetraploid individuals. This is not unexpected as observations from Newell and de Wet (1974) indicated that majority of eastern gamagrass in Illinois and the neighboring states is diploid (2n = 36) or tetraploid (2n = 72). They also demonstrated that phenotypic characteristics alone cannot be predictive of ploidy levels, even though tetraploid populations generally tend to be slightly more robust than diploid ones. Based on these observations it can be assumed that the populations utilized in the present study were comprised of diploid, tetraploid or a mixture of diploid and tetraploid individuals.

A total of 94,643 eastern gamagrass seeds were used in this study to test for either glyphosate-tolerance in the field screen or to evaluate for the presence of the *cp4 epsps* transgene in the molecular screen. The large sample size and different approaches were utilized to increase the probability of finding potential interspecific hybrids even if they occur at a very low frequency.

#### Presence/absence of glyphosate-tolerance in field screen

On average, 864 seeds from each of 54 eastern gamagrass populations were evaluated over a 2-year period in the field experiment (S-Fig. 2). Specifically, 21,450 seeds collected from 26 natural eastern gamagrass populations and 25,193 seeds collected from 28 natural populations were planted on the University of Illinois Research Farm in 2014 and 2015, respectively. Emergence rate averaged across populations was 45.2% in 2014 and 11.3% in 2015. A total of 12,524 emerged seedlings across both years were treated with the glyphosate herbicide. No seedlings survived the herbicide treatment (Table 2), which indicated a lack of glyphosate-tolerance across these diverse natural populations of eastern gamagrass. The absence of glyphosate-tolerance suggests a lack of interspecific hybridization between maize and eastern gamagrass even though these eastern gamagrass populations grew near commercial maize fields and were exposed to transgenic maize pollen for years.

**Table 2:** Field screen: mortality (%) of eastern gamagrass plants after glyphosate application

Year	Populations	Number of planted seeds	Number of emerged seedlings	Mortality (%)
2014	26	21,450	9687	100
2015	28	25,193	2837	100
Total	54	46,643	12,524	100

#### Presence/absence of cp4 epsps transgene in molecular screen

In order to evaluate a large number of eastern gamagrass seeds for the presence of the *cp4 epsps* transgene, method was developed to assess the sensitivity of a genotyping assay with regards to the number of seeds that can be bulked together. Results of the genotyping assay indicated that the assay is sensitive to reliably detect *cp4 epsps* in one out of 200 bulked seeds (Fig. 1). Thus, the decision to evaluate bulks of 50 seed in this study provided even higher assay sensitivity and additional certainty of detecting a very small *cp4 epsps* presence.

A total of 48,000 seeds from 54 populations of eastern gamagrass were tested for the presence/absence of *cp4 epsps* transgene. The genotyping was conducted on 960 samples of 50-seed pools. Each sample indicated the absence of glyphosate-tolerance *cp4 epsps* transgene. Initial testing showed that two out of 960 samples had an uncallable reading. These two samples were shipped to the Marker Discovery and Development laboratory at Monsanto for further investigation. Using the Endpoint TaqMan^®^ method these samples were found to be negative for *cp4 epsps*. A follow up experiment using real-time PCR was conducted, and the results indicated that the CP4 EPSPS signal was below the 1:400 control samples which was consistent with a very low level contaminant or non-specific amplification. These results indicated that all 48,000 tested seeds, representing 54 eastern gamagrass populations collected from 43 locations, showed an absence of *cp4 epsps* transgene (Table 3).

**Table 3:** Molecular screen: absence of *cp4 epsps* transgene in eastern gamagrass seeds

Collection years	Populations	Number of tested seeds	Number of samples	*cp4 epsps* detection (%)
2013/2014	54	48,000	960	0

Taken together, our results from the field screening and molecular genotyping show that no gene flow occurred from transgenic maize to eastern gamagrass even though the evaluated populations of eastern gamagrass have been exposed to CP4 EPSPS maize pollen over a 15-year period. These results are in agreement with observations from others researchers regarding limitations associated with outcrossing between maize and eastern gamagrass. First, experimental hybrids between maize and *Tripsacum* have only been obtained utilizing specific greenhouse and laboratory techniques (Mangelsdorf and Reeves 1931; Beadle 1980; Bernard and Jewell 1985; Randolph 1970; James 1979). Secondly, natural interspecific hybrids between maize and the *Tripsacum* genus have not been reported, even though *Tripsacum* grows in close proximity to millions of hectares of maize in the USA Corn Belt (Beadle 1980; Randolph 1970; Mangelsdorf and Reeves 1931). Talbert et al. (1990) conducted a cytogenetic study that showed that transposable elements (*Mu* and *Spm*) found in maize were absent from eastern gamagrass which provided additional evidence of lack of ongoing gene flow between the two species. Furthermore, no evidence exists of ongoing natural introgression between maize and the genus *Tripsacum* in South America (de Wet et al. 1972). Thirdly, lack of observed hybrids in nature is not surprising considering the complete male sterility observed in experimental hybrids (Beadle 1980; Randolph 1959; Talbert et al. 1990) and the genetic incompatibility between *Tripsacum* and maize due to irregular chromosome pairing (Beadle 1980).

### Assessment of interspecific hybridization between maize and eastern gamagrass

To further assess the possibility of hybridization and the production of viable offspring between maize and eastern gamagrass, hand cross-pollination attempts were conducted in greenhouse. Considering that diploid cytotype of eastern gamagrass reproduced sexually like maize, it was important to verify that the attempted cross-pollination was conducted with diploid rather than tetraploid eastern gamagrass plants whose reproduction is based on facultative apomixes. To minimize assessing asexually formed seeds, it was necessary to confirm the ploidy level of all eastern gamagrass plants used in the greenhouse experiment. Flow cytometric analysis confirmed that all eastern gamagrass plants used in attempted cross-pollination experiment were diploid (S-Fig. 3). The ploidy level determined by flow cytometric analysis was confirmed with chromosome counts. As expected for diploid cytotype, all eastern gamagrass plants used for attempted cross-pollination with maize had 36 chromosomes (S-Fig. 4).

Across the 2 years, 139 cross-pollinations were attempted between eastern gamagrass and maize (Table 4). Each eastern gamagrass inflorescence was composed of 1–3 spikes and produced 5–23 spikelets. In 2014, a total of 58 cross-pollinations were attempted. Among the 662 spikelets obtained, only 26 contained seed (all from different inflorescences). The 26 seeds were subjected to germination, but only 4 seedlings were obtained, while 22 of them did not germinate. Tissues from all 26 individuals (4 seedlings and 22 non-germinated seeds) were tested for the presence of the glyphosate-tolerant trait and all indicated absence of *cp4 epsps* transgene. Furthermore, there was no difference in genome size between maternal parent (eastern gamagrass) and the four seedlings, indicating lack of cross-pollination with maize.

**Table 4:** Greenhouse experiment: Success of interspecific pollination (EG^a^ × Maize) compared to intraspecific pollination (EG × EG)

Year	Pollination type	Number of pollination attempts	Number of spikelets^b^	Number of seeds	Number of spikelets per inflorescence	Pollination rate (%)^c^
2014	EG × EG	10	102	83	10.2	81.4
	EG × Maize	58	662	26	11.4	3.9 (0.0)
2015	EG × EG	10	108	86	10.8	77.8
	EG × Maize	81	867	1	10.7	0.1 (0.0)
Total	EG × EG	20	210	169	10.5	80.5
	EG × Maize	139	1529	27	11.0	1.8 (0.0)

^a^EG = eastern gamagrass used as maternal parent

^b^A total number of spikelets that were exposed to maize pollen (EG × Maize) or eastern gamagrass pollen (EG × EG)

^c^Pollination rate is expressed as percentage of spikelets with seeds. The number in parenthesis indicates the percentage of EG × Maize cross-pollinations

In 2015, a total of 81 cross-pollinations were attempted. Among the 867 spikelets obtained, only one seed was formed, but it did not germinate. In contrast, sib-pollinated eastern gamagrass resulted in high number of seeds as pollination rate was over 80% across both years (Table 4). This confirmed self-compatibility of eastern gamagrass and appropriateness of pollination techniques used in this study.

These 27 seeds harvested from eastern gamagrass plants across 2 years could not have resulted from outcrossing with maize, as *cp4 epsps* transgene from maize pollen was absent. These seeds could have been developed parthenogenetically, which has been observed with diploid eastern gamagrass when fertilization was absent (de Wet 1991). Alternatively, these seeds resulted from accidental, unintended self-pollination. Even though attempts were made to remove male portions of the spikes (S-Fig. 1), some male spikelets on the distal end of the female flower might have been overlooked resulting in a few self-pollinations. More seeds were observed in 2014 with fewer pollination attempts than in 2015 (26 and 1, respectively). In 2015, to further eliminate the possibility of self-pollination, the male inflorescence structures and the distal end of female flower structure were removed from the spikes, which resulted in much lower parthenogenetic/self-pollinated seed number. In summary, across both years, a total of 1529 spikelets were collected, but most were barren as only 27 seeds were formed. None of the seeds resulted from cross-pollination with maize. Even under conditions that favor outcrossing, eastern gamagrass used as female parent did not hybridize with maize. This is in agreement with Mangelsdorf and Reeves (1939) and Randolph (1970) who reported that they were not able to obtain viable seeds by pollinating diploid *Tripsacum* with maize pollen.

To our knowledge this is the first study to evaluate directly the potential of pollen-mediated gene flow from transgenic maize to natural populations of eastern gamagrass. No evidence of gene flow from transgenic maize to eastern gamagrass in nature was observed even though the two species have grown in close proximity for years and have had ample opportunities for outcrossing. These results should be taken in consideration when the Agreement of Maize Center of Origin in Mexico is updated as part of environmental risk assessment regarding the potential for pollen-mediate gene flow from transgenic maize to its wild relatives.

## Electronic supplementary material

Below is the link to the electronic supplementary material.

Supplementary material 1 (DOCX 6041 kb)

## Footnotes

1
^®^ *Roundup* is a registered trademark of Monsanto Technology LLC.
