# Supplementary material for: Assessment of the potential for gene flow from transgenic maize (Zea mays L.) to eastern gamagrass (Tripsacum dactyloides L.)
Source: Transgenic Res. 2017 May 2;26(4):501–14. doi: 10.1007/s11248-017-0020-7 (PMC5504203; doi:10.1007/s11248-017-0020-7)
Supplement: Supplementary file 1 — Supplementary material 1 (DOCX 6041 kb) [file 11248_2017_20_MOESM1_ESM.docx]

**Supplementary information**

S-Table 1 Details regarding field screen of eastern gamagrass for the glyphosate-tolerance

| Year | Planting | Seedling Count | Herbicide Spray | Plant Mortality Record |
| --- | --- | --- | --- | --- |
| 2014 | April 23 | May 26 | May 29 | June 23 |
| 2015 | May 18 | June 19 | June 23 | July 20 |

S-Fig. 1 The reproductive morphology of eastern gamagrass: (A) Whole spike with male (staminate) and female (pistillate) inflorescence structures, (B) Female portion of spike with receptive stigmas and (C) Female flower covered with an isolation bag

S-Fig. 2 Field screen of eastern gamagrass for glyphosate-tolerance (active ingredient of *Roundup*^[[1]](#footnote-1)^® agricultural herbicides): (A) Before *Roundup*^®^ application and (B) After *Roundup*^®^ application


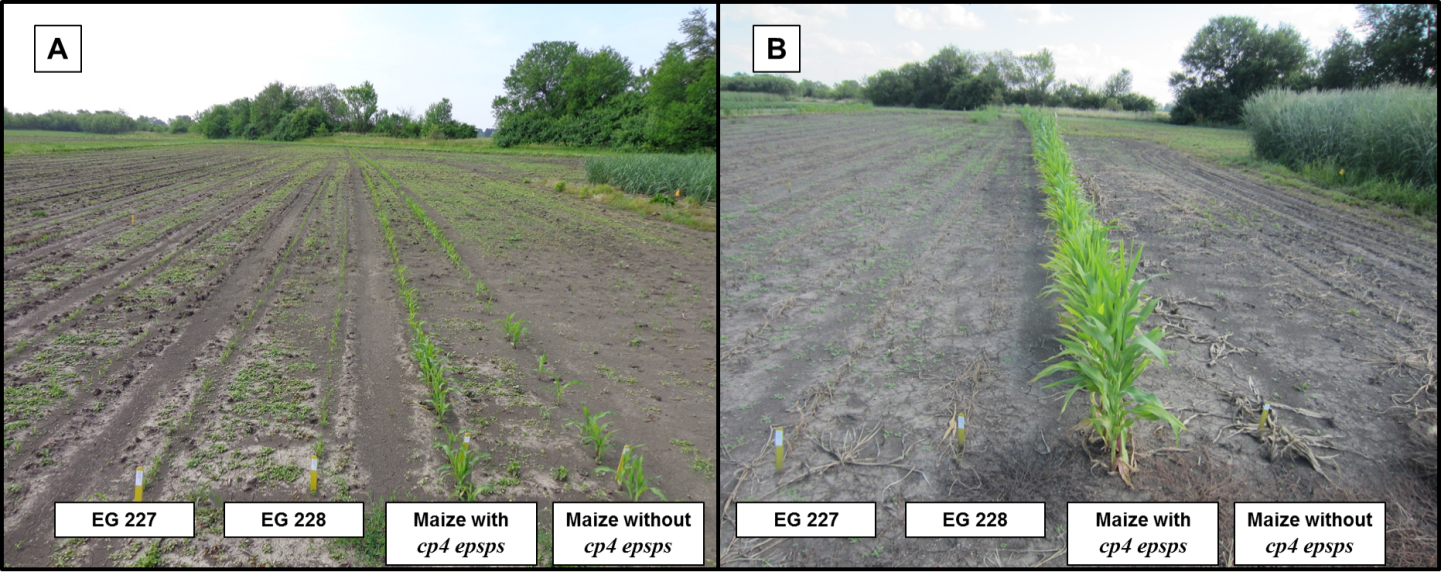


^†^EG227 and EG228: Examples of eastern gamagrass populations (*Tripsacum* *dactyloides* L.) collected in Illinois (Table 1).

S-Fig. 3 Flow cytometric histograms of maize and eastern gamagrass: (A) Diploid maize histogram, (B) Diploid eastern gamagrass, and (C) Tetraploid eastern gamagrass. The bars represent the number of nuclei used to estimate the mean fluorescence of each peak. Peaks of G1-2x and G1-4x indicate the G1 somatic nuclei of diploid and tetraploid eastern gamagrass, respectively, and G1- and G2-Maize represent the G1 and G2 nuclei of maize as an internal control co-homegenized with eastern gamagrass.

*
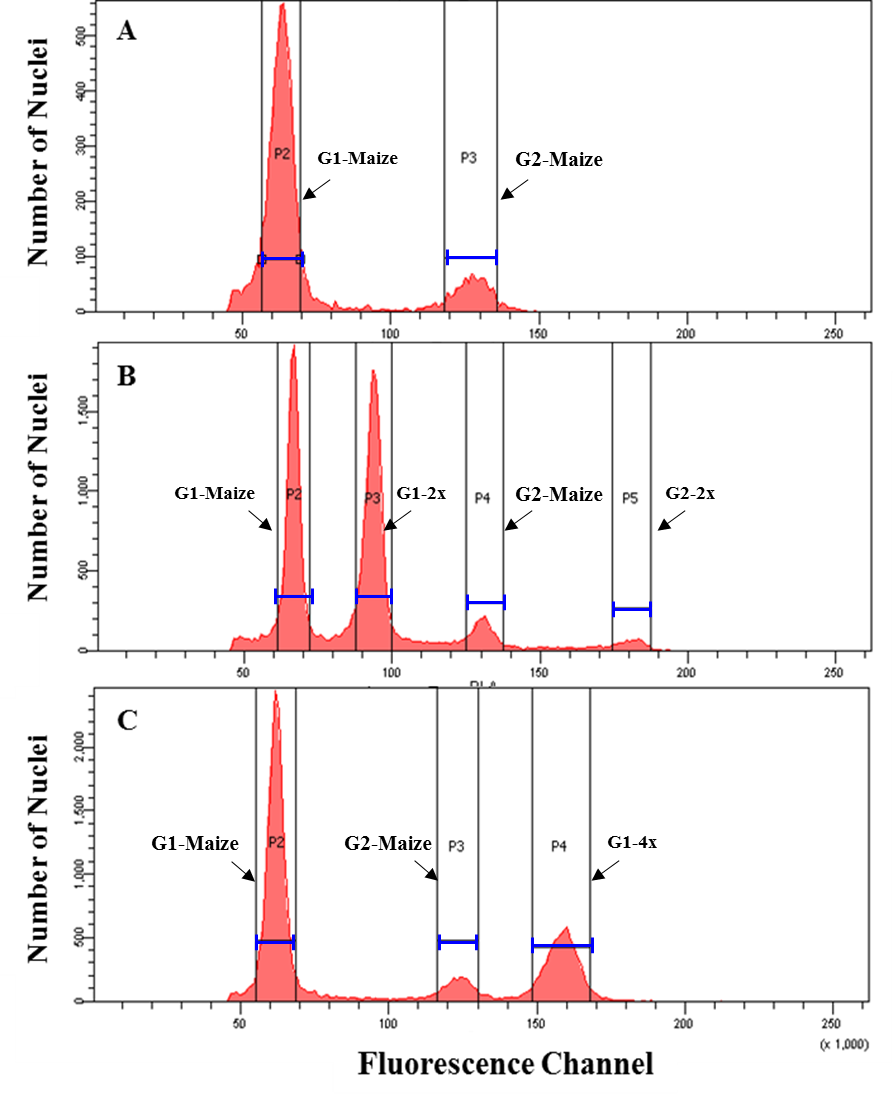
*

S-Fig. 4 Chromosomes (1-36) from the root tip cell of a diploid eastern gamagrass (2n=2x=36) imaged with a 60x magnification


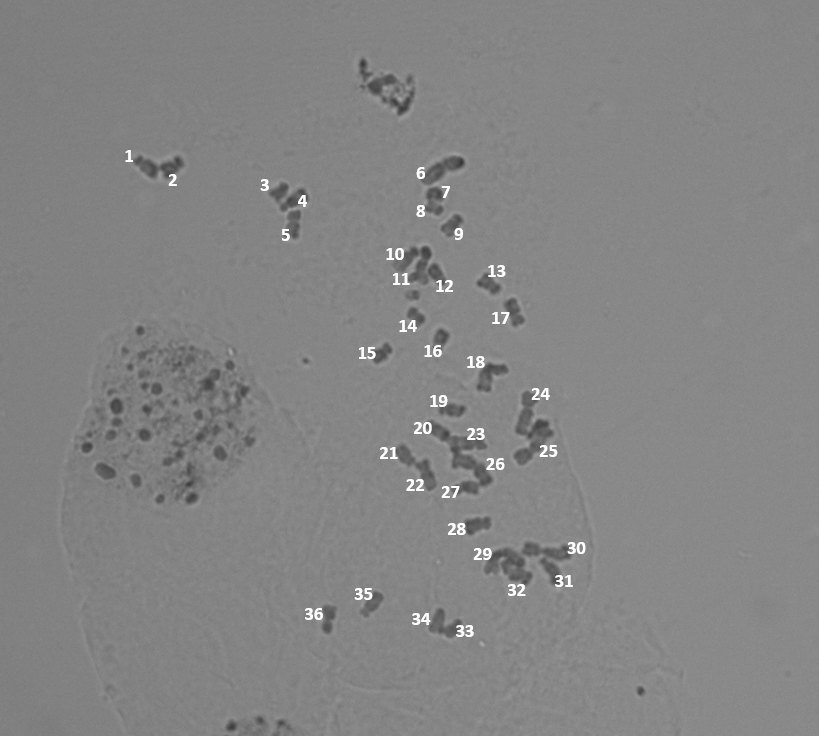


1. ® *Roundup* is a registered trademark of Monsanto Technology LLC. [↑](#footnote-ref-1)
